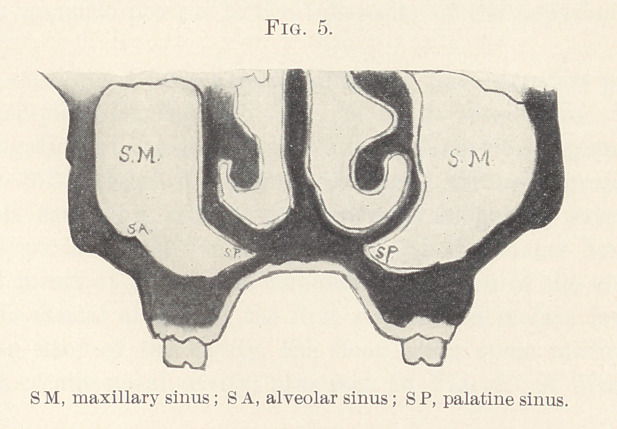# Development of the Maxillary Sinus

**Published:** 1901-04

**Authors:** Emma E. Musson

**Affiliations:** Philadelphia


### International Dental Journal.

Vol. XXII. April, 1901. No. 4.

### Original Communications.[1]

### DEVELOPMENT OF THE MAXILLARY SINUS.[2]

BY EMMA E. MUSSON, M.D., PHILADELPHIA.

Ancient historical literature relating to theories advanced on the anatomy and function of the pneumatic sinuses cannot fail but to be interesting to those working in these regions, though, curiously enough, the anatomists of ancient times seemed to have been better acquainted with the frontal and sphenoidal sinuses than with the more conspicuous and easily accessible antral cavities.

Some of the earlier anatomists of the sixteenth and seventeenth centuries proved to their satisfaction that the cavities of the frontal and sphenoidal bones were lined with a green membrane; later the statement was applied to the antrum, and in addition it was announced that these sinuses were filled with a medullary substance; another theorist advanced the idea that the function of this so-called medullary substance was to supply nutrition to the surrounding bones and to the teeth of the superior maxilla.

[1] The editor and publishers are not responsible for the views of authors of papers published in this department, nor for any claim to novelty, or otherwise, that may be made by them. No papers will be received for this department that have appeared in any other journal published in the country.

[2] Read before the Academy of Stomatology, Philadelphia, January 22, 1901.

Later authors combated the theory of the sinuses being filled with a medullary substance and contended that they were filled sometimes with mucus and sometimes with air, and a few were inclined to think that these spaces were an outlet for the fluids of the brain. Among a few authors who at this time advanced the theory that the sinuses were filled with air, or, in other words, were empty, was N. Highmore, after whom the antrum of the superior maxilla is named, who in a disquisition, written in 1681, combated the older theories.

In spite of this the anatomists still did not accept the theory of the sinuses as air-spaces, and Vieussens adhered to the old theory that they contained mucus, and that the function of these sinuses was to disembarrass the blood of mucus on its upward course to the brain. Anatomists now explain the green membrane by post-mortem changes and the presence of mucus, and a medullary substance by an ante-mortem catarrhal or purulent accumulation in the sinuses.

The theories as to the physiology of the air sinuses are equally interesting; probably the most ingenious was the one that they generated air and expurgated the animal spirits. In 1776 they were considered as necessary to phonation, later still to olfaction, the antrum excepted; though Vesalius had, before 1766, seen in these cavities a formation of bony structure that combined lightness and volume, the theory of the present day.

According to Durcy, the first outlines of the maxillary sinus is represented by a lateral invagination (depression outward) of the nasal mucous membrane, corresponding to an excavation in the rather thick wall of the cartilaginous capsule of the nose. Later this cartilaginous capsule becomes surrounded by bony tissue and, disappearing, the mucous membrane of the sinus is lodged in a bony diverticulum. At four and five months we have the antrum developed as in Figs. 1 and 2.

In the new-born the maxillary sinus is a small depression posterior to the lachrymal duct at the level of the second molar (Fig. 3); in the second year the amount of space between the infraorbital canal and the canine milk-tooth is ten millimetres. At eight and nine years of age it has extended up into the zygomatic apophysis, and in its transverse diameter has taken definite shape. With the descent and eruption of the permanent teeth, the depth and the height of the sinus is increased. The

Fig. 1.

S, ethmoidal mass; P, unciform process; m, inferior turbinate.

Fig. 2.

S, ethmoidal mass; B, bulba ethmoidalis; P, unciform process; m, inferior turbinate.

Fig. 3.

S M, maxillary antrum.

Fig. 4.

S M, maxillary sinus; S A, alveolar sinus; P A, alveolar process.

Fig. 5.

S M, maxillary sinus; S A, alveolar sinus; S P, palatine sinus.

dimensions are, therefore, never fixed until after the end of the second dentition.

Macrosmatique mammals, those with the sense of smell highly developed, as in dogs, have open maxillary sinuses as seen in the Plate, and form simply a notch which is large posteriorly at the expense of the palatine bone. The orang-outang has one large single cavity formed by the maxillary sinus and ethmoidal cells, this communicating by a large, free opening with the sphenoidal sinus, as seen in the Plate.

There is the greatest variety in the size and shape of the antrum; the process of resorption of the bony tissue may go on to its full extent, leaving a large sinus with thin walls, or the process becomes inhibited with the result of a small sinus with thick, bony walls; or, again, the development of the walls of the antrum is defective. There, however, always seems to be a more or less constant relation between the development of the nasal chambers and that of the antral cavities,—a wide nasal cavity, a narrow antrum, and *vice versa*. Zuckerkandl's table of normal measurements is as follows: Frontal dimensions of air-spaces in the superior maxillary, sixty-eight millimetres; width of the nasal fossa, thirty-one millimetres; height of antrum, twenty-six millimetres. In a case of atrophy of the antrum: dimension of air-space, sixty-nine millimetres; width of the nasal fossa, forty-eight millimetres; height of right antrum, twenty-two millimetres; height of left antrum, eighteen millimetres; a difference of seventeen millimetres in increase of width of the nasal fossa and a corresponding diminution in that of the antral cavity. In taking height of antrum, its nasal wall is considered as the base, the apex being a line running from the zygomatic process to the tuberosity of the superior maxilla.

In the specimen I have here this evening the resorptive process has been complete, leaving thin walls for all its boundaries. There is a prolongation of the sinus up into the infraorbital region, producing an infraorbital fossa, and causing a strongly projecting infraorbital canal; a second and third prolongation extending into the zygomatic and frontal processes of the malar bone; a fourth the result of the almost complete resorption of the alveolar process, the dental alveoli of the first and second molars forming part of the floor of the cavity, the floor being some millimetres below that of the nasal cavity, also seen in Fig. 4. A fifth prolongation is the extension of the fossa between the plates of the palatal portion of the superior maxilla, almost as far as the spinous process,—that is, the middle line, also seen in Fig. 5. Thus, the upper wall of the sinus to a large extent forms the floor of the nose. The width of the nasal fossa measures twenty millimetres, the height of the antrum forty-three millimetres, and the depth forty millimetres. Zuckerkandl gives forty-six millimetres as the greatest width of antrum found in his dissections. Such an antrum is to be diagnosed in life in several ways: the narrowness of the nasal chamber or lack of bulging outward of external nasal wall; the lack of depression of the facial wall of the antrum; the bulging outward of the antrum posterior to the zygomatic process; and by the transillumination test, the brilliant illumination of the infraorbital fossæ and the pupils.

Surgically such an antrum can be easily opened at any point the operator should choose to elect, even from the roof of the mouth; also in the radical operation in the canine fossa the surgeon would run no risk of wounding the infraorbital nerve, so great is the height of the sinus from alveolus to the infraorbital canal.

Incomplete resorption, or arrested development, and approximation of the maxillary walls are the chief causes of stenosis of the antrum. To these may be added depression of the canine fossa, thickening of the walls of the antrum, and bulging of the external nasal wall into the antrum.

When there is incomplete resorption of bone, we have, taking the place of the normal fossa, a finely cancellated bone-tissue; this lack of absorption is mainly confined to the alveolar process, in consequence of which the floor of the antrum will be some six to nine millimetres above the floor of the nose, and thus between the roots of the molars and the floor of the antrum there is a thick layer of bone which renders the drilling into the antrum a painful, tedious process; for the same reason, drilling through the inferior meatus would be difficult if not impossible. In such cases the canine fossa would be indicated as the point of entrance.

In approximation of the maxillary walls, examination would demonstrate the depressed facial wall and a bulging outward of the external nasal wall. Depression outward of the nasal wall is almost always at the expense of the antrum, and may take place either at its lower or its upper portion, depending whether the depression is found in the inferior or middle meatus. This conformation is very readily diagnosed by a nasal examination, which reveals a deeply concave inferior or middle meatus; the latter condition is very well shown in these specimens. The surgical importance of this variety of stenosis would not be the difficulty of penetrating into the antrum, but the danger of going through both walls and into the nasal cavity.

A condition of antrum impossible to foresee is that of its division into two sections by a partition wall. In the plate presented the sections are separated by a vertical division wall, and the sinuses thus formed communicate separately with the middle meatus. (Fig. 4.)

Only in the neighborhood of the maxillary ostium does the mucous membrane resemble the pituitary membrane of the nose; in its greatest extent it is pale and thin, and easily separates into two layers. The superficial layer, the so-called mucous membrane proper, is ciliated, and contains but few glands; the deep layer is adherent to the bone beneath and takes the place of the periosteum. The interval between these layers is occupied by a very loose cellular tissue which contains a large number of glands; this latter becomes markedly swollen and infiltrated in the course of an inflammatory process in the antrum, and the whole mucous membrane, thin and pale as it is, will in the course of a short period become transformed into a thick, fungous tissue, with a tendency to the formation of polypoid growths.

On the anterior and lateral walls of the antrum will be found numerous canals containing the dental nerves and their accompanying blood-vessels; their walls are at many points imperfect, and thus the nerves lie in direct contact with the lining mucous membrane of the antrum and will be subject to all of the inflammatory conditions of this membrane, giving rise to the severe attacks of pain present in acute antral sinusitis.

## Figures and Tables

**Fig. 1. f1:**
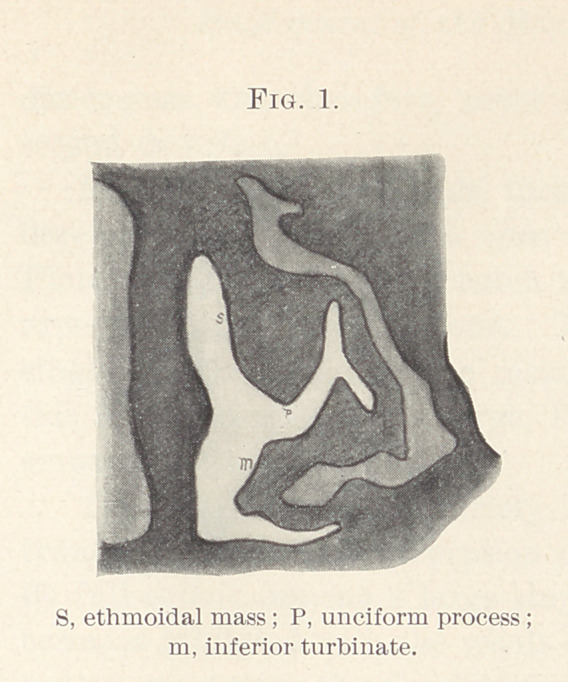


**Fig. 2. f2:**
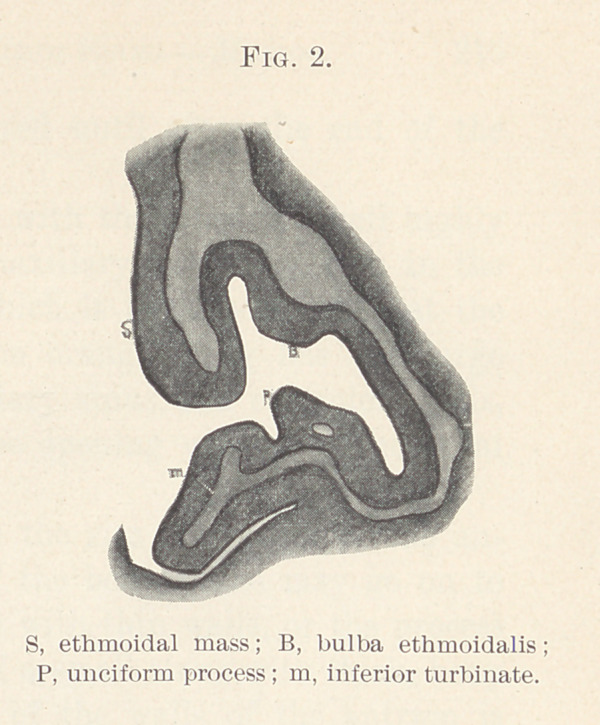


**Fig. 3. f3:**
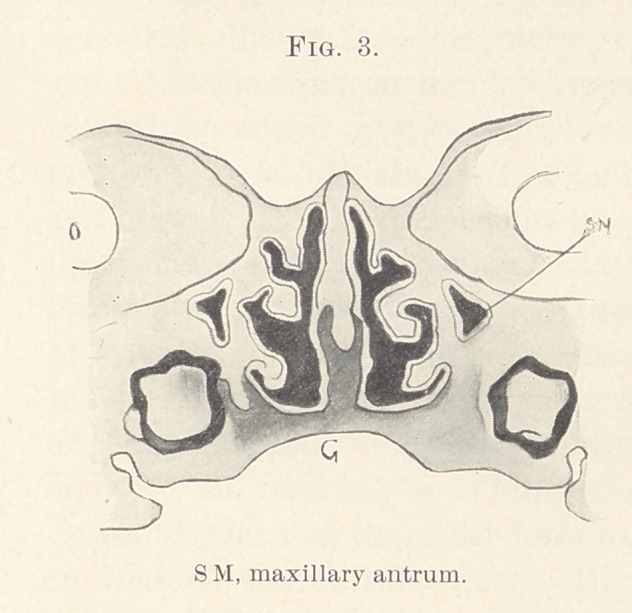


**Fig. 4. f4:**
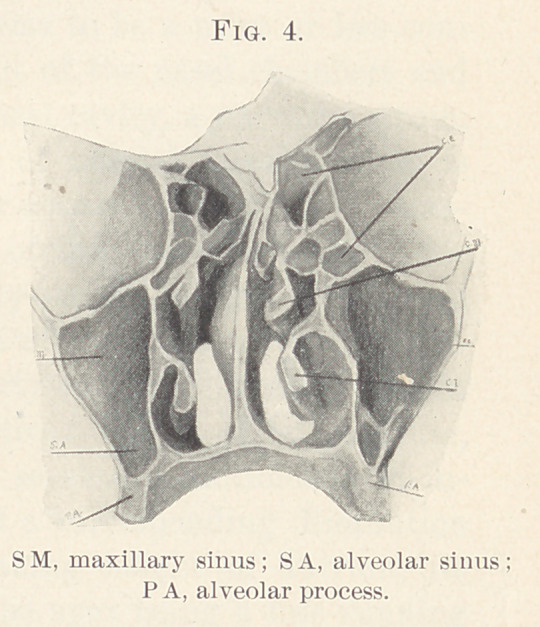


**Fig. 5. f5:**